# A Rare Cause of Recurrent Left Knee Pain in an Adolescent Male: A Case Report of Distal Femoral Brodie's Abscess

**DOI:** 10.1002/ccr3.70644

**Published:** 2025-07-27

**Authors:** Adeel Ahmed Siddiqui, Muhammad Waqas Khan, Sajjad Ahmed, Taimoor Ali, Asif Ali, Hussain Haider Shah, Tirth Dave

**Affiliations:** ^1^ Department of Orthopaedic Surgery Dow University of Health Sciences Karachi Sindh Pakistan; ^2^ Bukovinian State Medical University Chernivtsi Ukraine

**Keywords:** acute leg pain, atraumatic limp, Brodie's abscess, chronic osteomyelitis, subacute osteomyelitis

## Abstract

Brodie's abscess, a subacute form of osteomyelitis, is characterized by localized symptoms and can be challenging to diagnose due to its nonspecific clinical presentation. We report a rare case of distal femoral Brodie's abscess in a healthy child, emphasizing the diagnostic intricacies and management. A 15‐year‐old previously healthy patient presented with chronic left knee pain of insidious onset and progressive nature. Conservative measures were initially employed, leading to transient symptom resolution. Clinical examination revealed limited flexion, and imaging demonstrated a well‐corticated osteolytic lesion with a sclerotic rim on the distal femur, along with the characteristic “penumbra sign” on magnetic resonance imaging (MRI). Brodie's abscess can mimic benign conditions, leading to delayed diagnosis. Clinicians should maintain vigilance when evaluating patients with unexplained joint pain, especially in the absence of systemic symptoms. Early recognition and appropriate management, as exemplified in this case, are crucial for preventing complications and ensuring a favorable outcome. This report contributes to the understanding of this rare condition and highlights the significance of timely intervention in Brodie's abscess cases.

**Abbreviations**
CRP: C‐reactive protein
ESR: erythrocyte sedimentation rate
MRI: magnetic resonance imaging

**Summary**

- This rare case report highlights the diagnostic challenges of Brodie's abscess, a subacute form of osteomyelitis.
- We present a case of a healthy child with chronic knee pain and describe the distinctive imaging features, emphasizing the importance of timely diagnosis and surgical intervention. Brodie's abscess can masquerade as benign conditions, leading to delayed treatment. Clinicians should maintain a high index of suspicion when evaluating patients with atypical joint pain.

## 1 Introduction

In terms of clinical course, bone infection can be classified as acute, subacute, or chronic osteomyelitis [1]. Brodie's abscess is an unusual form of subacute osteomyelitis distinguished by the buildup of pus within the bone. Typically, this condition exhibits a gradual start and presents with localized clinical signs, rather than widespread symptoms [2]. The term was named after Sir Benjamin Collins Brodie in 1832, who originally characterized the condition as a localized bone abscess that developed without any previous signs of systemic illness. In most cases, the microorganisms responsible for causing infections are *Staphylococcus aureus* , accounting for 80%–90% of cases, Streptococcus B in newborns, and Pseudomonas, which is more frequent among drug users. Individuals with type 2 diabetes or sickle cell disease may experience infections caused by Salmonella, while other common microorganisms include *Mycobacterium tuberculosis* , Spirochaetes, fungi (such as Candida and Actinomyces), viruses, and helminths (such as Echinococcus) [1]. *Haemophilus influenzae* , *Kingella kingae* . It is worth noting that in more than 50% of cases of Brodie's abscess, it is not possible to culture the organism.

Niels van der Naald et al. present the first systematic review of all published cases related to Brodie's abscess. Their study encompasses a comprehensive analysis of 70 aggregated studies, with a total of 407 patients, spanning the period from 1921 to 2018. The findings of this study shed light on the gradual development of Brodie's abscess, whereby the manifestation of systemic inflammatory indicators and symptoms is often absent. The condition was frequently observed in young males, with the tibia being the most commonly impacted bone. The primary form of treatment was surgery, which was followed by antibiotics in 77% of cases. Alternatively, in 17% of cases, only surgery was performed, reporting favorable outcomes [3].

Unfortunately, it is descriptive research, not an outcome study, and therefore confined to case reports, all observational and with considerable variability on reported items, which are also predominantly from highly developed countries and may thus present a deceiving representation. Its objective is to offer insight into the clinical features, treatment options, outcomes, and consequences of Brodie's abscess on the basis of case reports previously documented in the medical literature to date.

Diagnosing accurately and on time can be challenging as the most common symptoms are pain and swelling, which can be vague and stereotypical. Patients usually do not have a fever, and their inflammatory markers are normal and rarely show any other signs of systemic disease [4]. Since pain is not a physiological or hematological sign of illness, many cases are treated symptomatically until definitive testing and treatment can be done [5].

A five‐year retrospective research in south western Nigeria assessed 20 individuals with clinical and radiological signs of subacute osteomyelitis, with only 2.4% of the total cases caused by Brodie's abscess [6].

This case report is based on a patient who presented with symptoms similar to that of Brodie's abscess. What makes this case interesting is the extended duration of the abscess progression, which was caused by inadequate medical care and limited accessibility. This provides crucial insights into the challenges associated with diagnosing Brodie's abscess. The case report sheds light on the importance of timely and appropriate medical intervention in cases.

## 2 Case History/Examination

A 15‐year‐old patient in otherwise good health presents to our outpatient department with left knee pain. The patient mentions a one‐year history of intermittent episodes of knee pain with insidious onset and progressive evolution. The patient did not seek medical evaluation or treatment and instead relied on conservative measures to manage the pain, which ultimately led to the resolution of symptoms. Although there was no associated swelling or severe pain, the affected joint was stiff and exhibited trouble with ambulation. He denied the existence of any locking, catching, or clicking. He was afebrile at presentation and had normal vital signs (Temperature of 99.1°F). Before the onset of symptoms, he had not suffered trauma and had no prior medical or surgical history. His left knee was examined and found to have intact skin warmth to palpation, limited flexion up to 20°, which was painful, ligaments stable, and no erythema. The remainder of his examination was normal.

## 3 Methods (Differential Diagnosis, Investigations, and Treatment)

**FIGURE 1:**
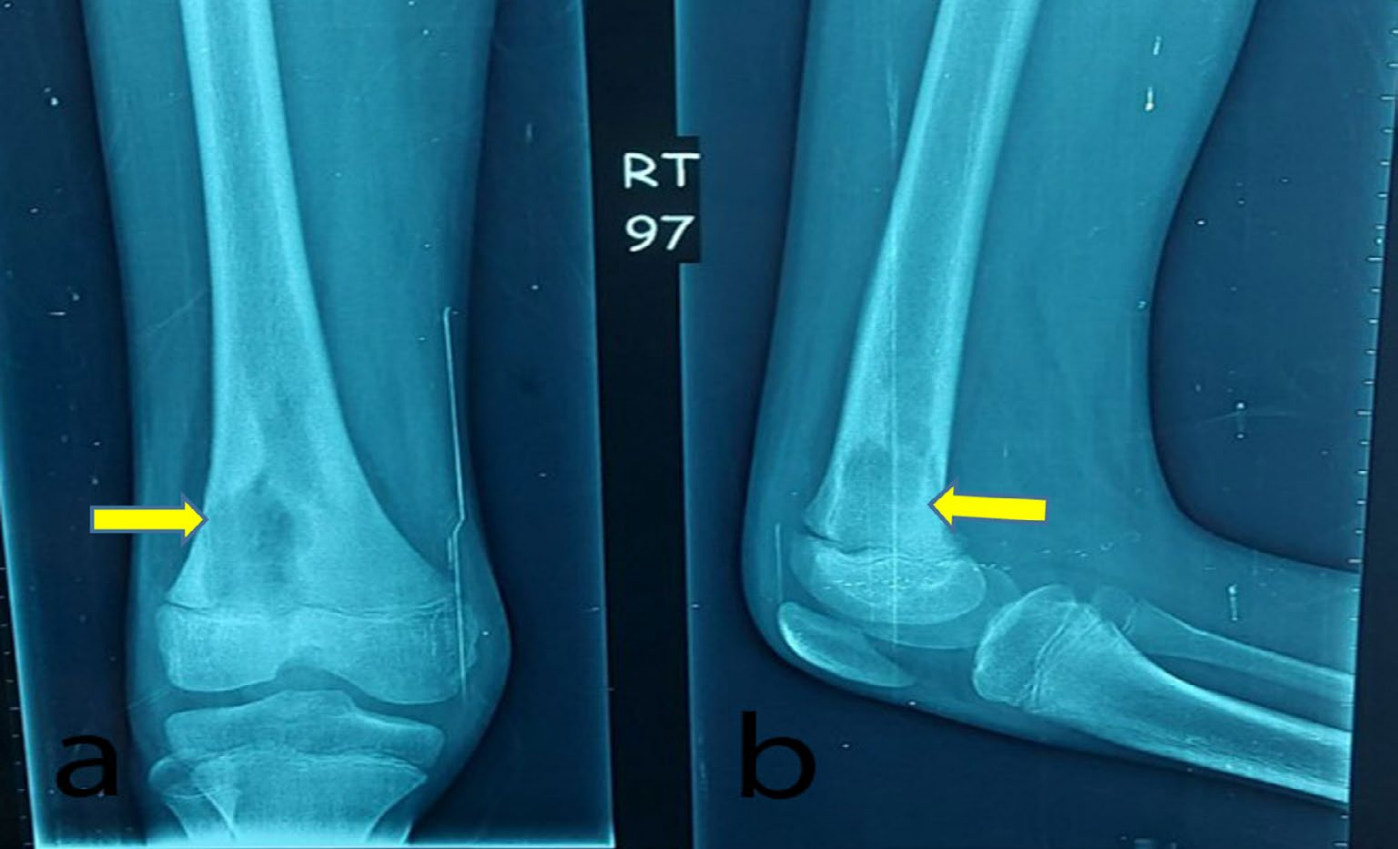
(a) Anteroposterior view showing well‐defined osteolytic lesion extending to epiphysis with sclerotic rim, (b) lateral view.

The patient was sent for knee X‐rays, which revealed a properly defined and well‐corticated osteolytic lesion with a sclerotic rim on the distal femur (Figure 1).

**FIGURE 2:**
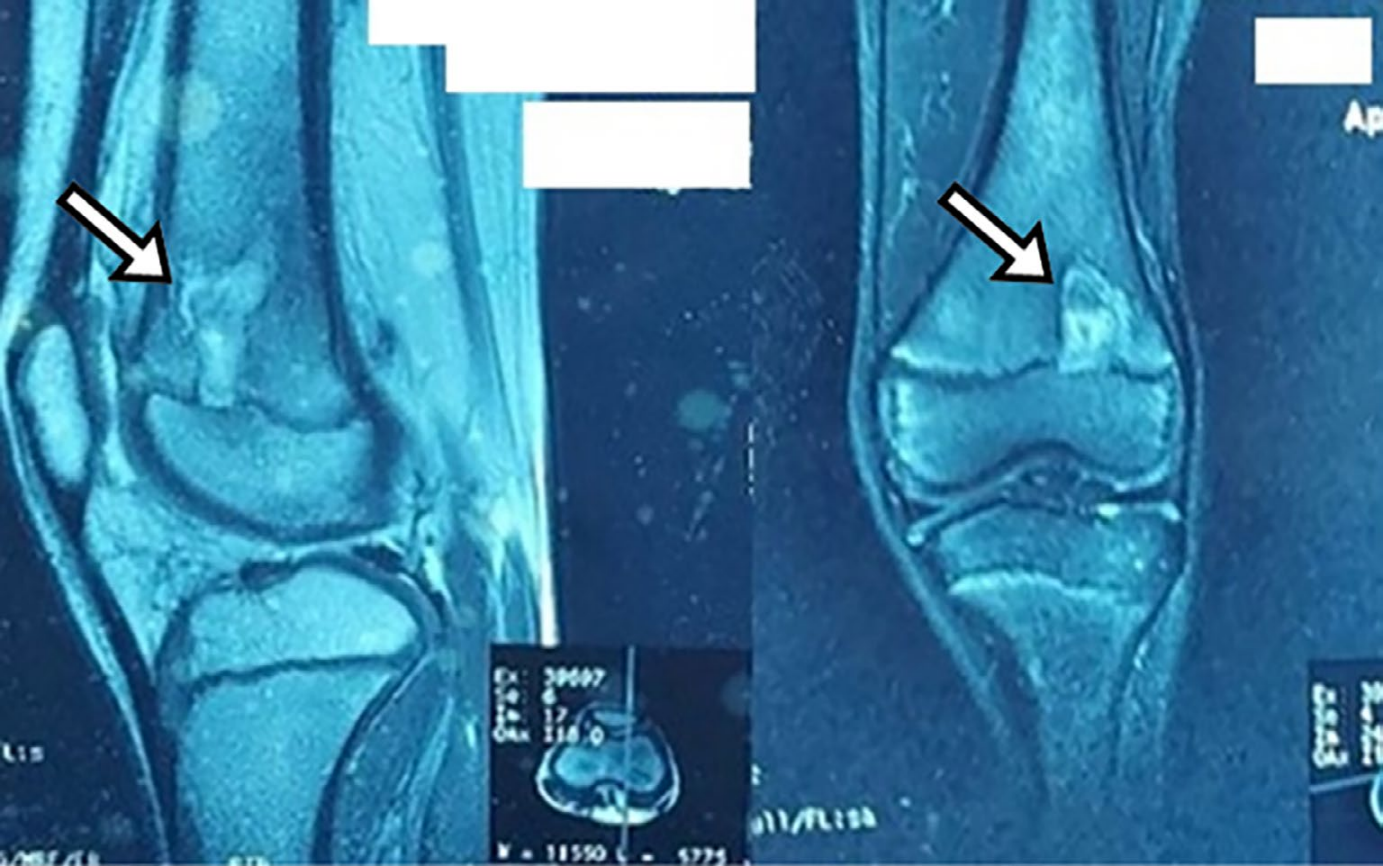
Contrast T1‐weighted images showing penumbra sign, in sagittal (Right) and coronal images (Left).

Magnetic resonance imaging (MRI) with contrast was performed to better characterize the lytic lesion. The MRI scan reveals the distinctive ‘penumbra sign’ seen on T1‐weighted images, which is a thin granulation tissue layer that forms a rim of abscess, characteristically seen in subacute osteomyelitis (Figure 2).

**FIGURE 3:**
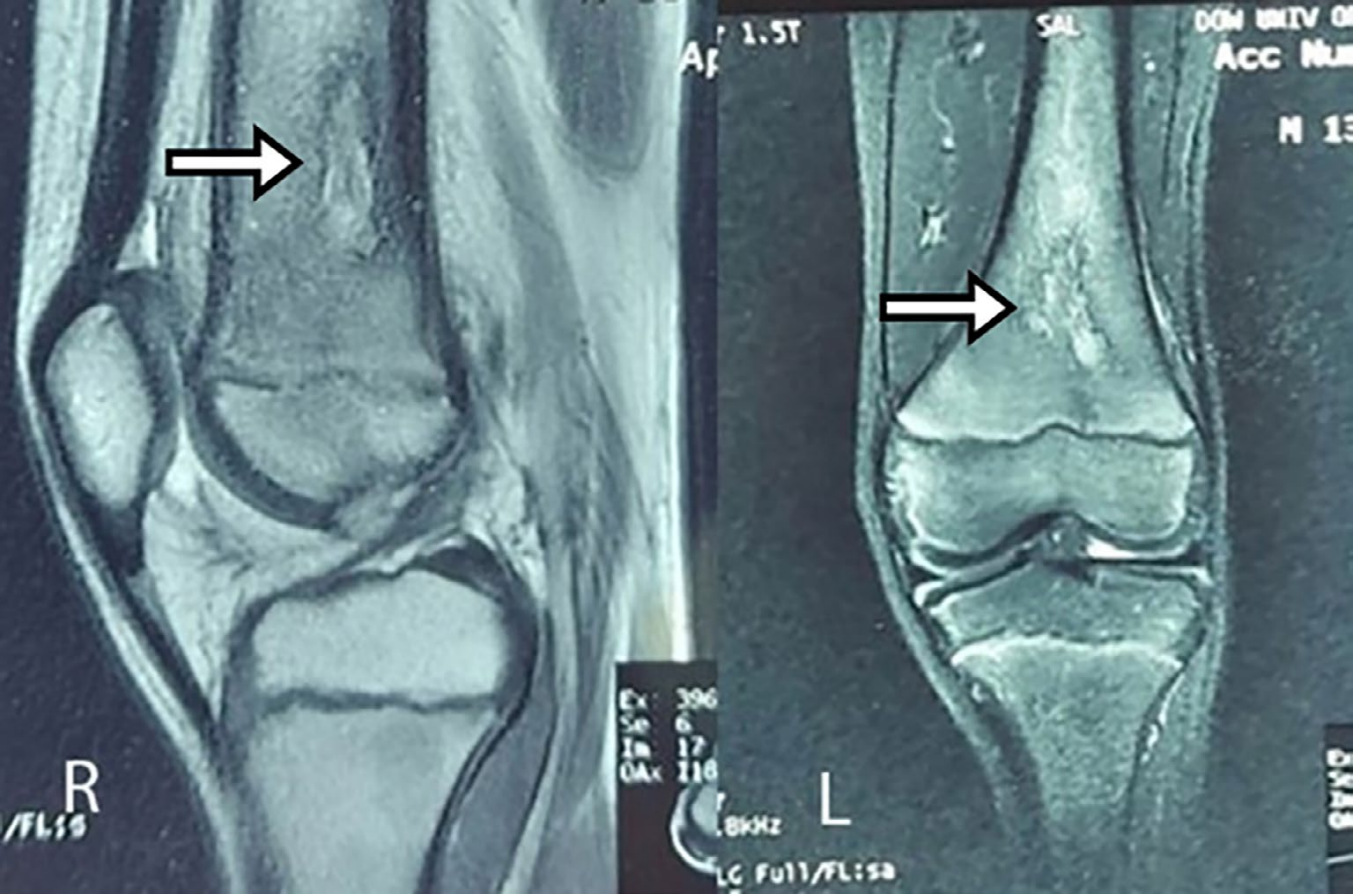
Heterogeneous on T2W1 and shows post‐contrast enhancement. Sagittal view (Right) and coronal view (Left).

It consists of linear abnormal signal intensity at the epiphyseal/metaphyseal junction of the left femur extending superiorly in the medullary cavity at the postero‐lateral cortical break that is hypo‐intense on T1 and heterogeneous on T2W1 (Figure 3) and shows post‐contrast enhancement.

## 4 Conclusions and Results (Outcome and Follow‐Up)

Mild edematous changes were identified at the level of the bone. The imaging finding called “penumbra sign” with a sensitivity of 75% and a specificity of more than 90% is a helpful imaging tool for the final diagnosis of subacute osteomyelitis [5].

The penumbra is a partial or lighter shadow cast by an eclipse. Subacute osteomyelitis is characterized by the presence of a delicate layer of granulation tissue that surrounds the abscess cavity [7, 8].

According to a study conducted by B. McGuinness et al., it has been observed that on unenhanced T1‐weighted imaging, the penumbra sign manifests as an area of relatively increased signal intensity. This region is situated between the abscess cavity, which exhibits intermediate to low signal intensity, and the surrounding bone marrow that may display edematous or sclerotic characteristics. The interobserver reliability of this particular assessment is generally considered to be moderate to good, as indicated by an average kappa value of 0.57. Additionally, it has been indicated that the penumbra sign exhibits a notable specificity of 96% but a limited sensitivity of 27% when it comes to musculoskeletal infections. This particular sign proves to be valuable in discerning neoplasia from infection [9].

Another study retrospectively reviewed the imaging investigations of 32 patients referred to an orthopedic oncology service, but subsequently proven to have osteomyelitis, and revealed that the T1‐W images of 24 (75%) patients displayed the penumbra sign [8].

**FIGURE 4:**
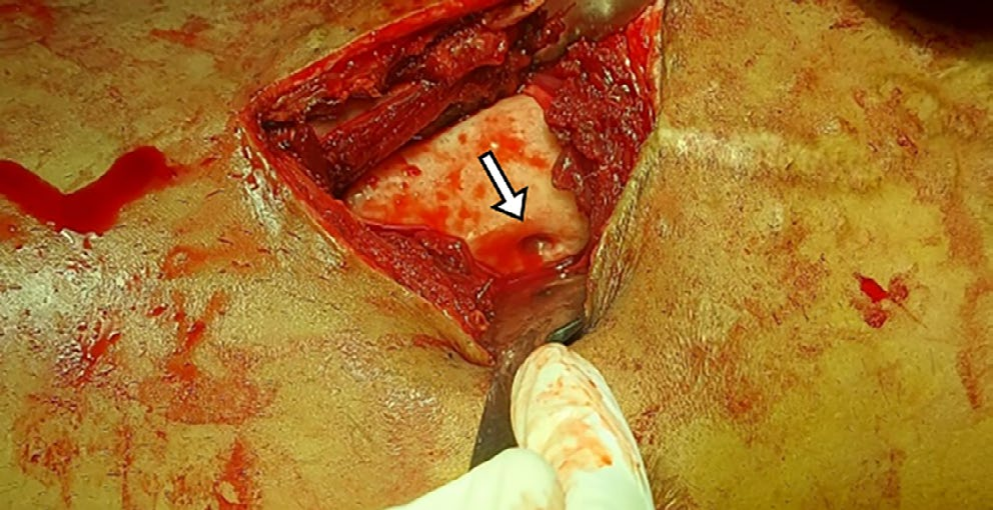
Intraoperative image showing draining sinus from posterolateral cortex of distal femur.

The patient was initially treated as an outpatient with analgesics. After a radiological review, he was admitted to the hospital, offered intravenous antibiotics, and was scheduled for elective surgery, during which the abscess was incised, drained, and sent for culture and sensitivity (Figure 4).

Bone was drilled with holes to increase blood flow. Curettage was performed, and 1 g of Vancomycin was injected into the cavity. The wound was then sutured with appropriate homeostatic measures. Following the surgical procedure, the patient experienced a seamless recovery process devoid of any complications. The pain experienced by the patient was effectively managed through the utilization of basic analgesic measures. The patient was initiated on a course of long‐term antibiotics, consisting of a two‐week intravenous administration during their hospitalization, followed by a two‐week regimen of oral doses upon their discharge. Furthermore, a subsequent appointment has been arranged for 6 weeks' time with the specialized pediatric orthopedic department.

The deep tissue specimens excised during surgery were submitted for Gram staining, AFB (acid‐fast bacilli) staining, and further histopathological evaluation to exclude tuberculosis. Intraoperative cultures indicated the presence of Klebsiella species.

## 5 Discussion

In English‐language literature, subacute osteomyelitis is often used to refer to a hypovirulent illness with a low‐grade clinical course. One example is Brodie's abscess, which can be present in various ways. This highlights the difficulty in distinguishing between subacute and chronic osteomyelitis. However, chronic osteomyelitis often results from an uncontrolled acute septic infection, while Brodie's abscess appears to be subacute from the beginning. Additionally, if the septic phase of acute osteomyelitis resolves without symptoms, subacute or chronic osteomyelitis may develop [1].

Brodie's abscess, often involving the metaphysis of long bones like the distal femur, is a localized form of subacute osteomyelitis. It is characterized by a walled‐off area of infection, typically surrounded by granulation tissue and reactive sclerosis. In the case of a Brodie's abscess in the distal femur, it is an uncommon location for this pathology, adding to its uniqueness. The rarity of this condition is highlighted by the fact that most literature on Brodie's abscess consists of only case presentations.

A systematic review of case reports on Brodie's abscess, covering studies published between 1921 and 2018, revealed that cases were documented globally, with the majority originating from the United States (16 cases), the United Kingdom (10 cases), and India (6 cases). The review included 70 studies encompassing a total of 407 patients. The median age of patients was 17 years (SD 11; based on 169 cases where age was reported). Brodie's abscess was more frequently observed in men, with a male‐to‐female ratio of 2.1:1 [3].

The most common complaint in most patients is pain, which is followed by a little loss of function or limping. Because the symptoms of a Brodie's abscess are sometimes ambiguous, a proper diagnosis is generally delayed, with the average length of symptoms ranging from 1 month to 2 years [2, 10, 11].

A study involving 1037 individuals with osteomyelitis discovered that all of them suffered from discomfort and localized swelling, but none of them had a fever, localized tenderness, or warmth [10]. Brodie's abscess is prevalent mostly in the metaphysis of the tibia or femur [12, 13]. Upper‐extremity abscesses are infrequent [12].

The presented case demonstrated a majority of the distinctive features associated with Brodie's abscess. The patient was a young boy who presented with nonspecific symptoms of pain and discomfort. It is surprisingly interesting that for more than a year, the patient complained of intermittent pain in his left lower leg, which led him to multiple visits to the general practitioners. The laboratory results appeared to be within the normal range, except for a slightly raised ESR and CRP. The plain radiograph followed by MRI findings highly suggests Brodie's abscess.

Plain radiography reveals a lytic lesion with a peripheral sclerotic ring receding in Brodie's abscess [14]. The unique characteristics of MRI prove to be valuable in the diagnostic assessment of Brodie's abscess. Marti‐Bonmati et al. were the pioneers in elucidating the characteristic appearance of MRI known as the “target” sign. This radiological finding comprises four discernible layers: (1) a central core, (2) two concentric rings, and (3) an outer peripheral halo. The abscess cavity is characterized by a central region. The inner ring corresponds to the granulation layer, while the outer ring signifies reactive sclerosis [8, 15].

MRI has proven superior to plain X‐ray in adults for diagnosing osteomyelitis. It is also the modality of choice in the pediatric population for discerning between infection and bone tumors [10].

Brodie's abscess radiological findings are comparable to those of other benign and malignant bone lesions that cause inflammation in the surrounding area, such as osteoid osteoma, Langerhans cell histiocytosis, chondrosarcoma, Ewing's sarcoma, and traumatic lesions [9, 13, 14, 16, 17].

The best course of treatment relies on an accurate diagnosis, followed by prompt, aggressive surgical debridement, defect repair [3], and long‐term antibiotics [4].

Curettage, biopsy, and culture of the lesion, followed by 6 weeks of antibiotic therapy, or the use of impregnated antibiotic beads, cancellous bone grafting following curettage, followed by immobilization, and antibiotic alone without surgery are all options for treating Brodie's abscess [6].

According to many authors, surgery remains the cornerstone of treatment. Antibiotics should be administered following drainage and collection of samples for culture and cytology/histology [3].

It has previously been proposed that surgery be reserved for aggressive lesions with ESRs more than 40 mm/h. It was also proposed that only individuals with abscesses larger than 3 cm be operated on [6].

In our case, we performed surgical treatment because the abscess was chronic, and it has been shown that surgical debridement followed by post‐operative antibiotics reduces medical expenditure, length of hospital stay, and rate of complications compared to treating with a conservative course of antibiotics alone [5, 10, 18]. One of the therapeutic approaches for Brodie's abscess is antibiotic‐impregnated beads [5] and antibiotic‐loaded cement spacers [19]. Nevertheless, we believe that removing the bony sequestrum and radical curettage are the most crucial approaches for the surgical procedure.

Due to its low incidence rate, nonspecific clinical features, and the absence of systemic symptoms, Brodie's abscess may often go unnoticed by physicians. However, in order to prevent complications and the possibility of long‐term disability, it is imperative that clinicians exercise extreme caution in evaluating pediatric or young adult patients who present with atraumatic limb pain.

## Author Contributions

**Adeel Ahmed Siddiqui:** conceptualization, supervision, writing – original draft, writing – review and editing. **Muhammad Waqas Khan:** writing – original draft, writing – review and editing. **Sajjad Ahmed:** writing – original draft, writing – review and editing. **Taimoor Ali:** writing – original draft, writing – review and editing. **Asif Ali:** writing – original draft, writing – review and editing. **Hussain Haider Shah:** supervision, writing – original draft, writing – review and editing. **Tirth Dave:** supervision, writing – original draft, writing – review and editing. **Adeel Ahmed Siddiqui, Muhammad Waqas Khan, Sajjad Ahmed, Taimoor Ali, Asif Ali, Hussain Haider Shah and Tirth Dave:** drafting, editing, revising, and finalizing the case report.

## Ethics Statement

Ethical approval was not required for the case report as per the country's guidelines.

## Consent

Written informed consent was obtained from the patient to publish this report.

## Conflicts of Interest

The authors declare no conflicts of interest.

## Data Availability

The data that support the findings of this article are available from the corresponding author upon reasonable request.
